# Functional Activation in the Ventral Object Processing Pathway during the First Year

**DOI:** 10.3389/fnsys.2015.00180

**Published:** 2016-01-05

**Authors:** Teresa Wilcox, Marisa Biondi

**Affiliations:** Infant Cognition Lab, Department of Psychology, Texas A&M UniversityCollege Station, TX, USA

**Keywords:** infants, object processing, object processing pathway, ventral temporal cortex, cortical development

## Abstract

Infants' capacity to represent objects in visual working memory changes substantially during the first year of life. There is a growing body of research focused on identifying neural mechanisms that support this emerging capacity, and the extent to which visual object processing elicits different patterns of cortical activation in the infant as compared to the adult. Recent studies have identified areas in temporal and occipital cortex that mediate infants' developing capacity to track objects on the basis of their featural properties. The current research (Experiments 1 and 2) assessed patterns of activation in posterior temporal cortex and occipital cortex using fNIRS in infants 3–13 months of age as they viewed occlusion events. In the occlusion events, either the same object or featurally distinct objects emerged to each side of a screen. The outcome of these studies, combined, revealed that in infants 3–6 months, posterior temporal cortex was activated to all events, regardless of the featural properties of the objects and whether the event involved one object or two (featurally distinct) objects. Infants 7–8 infants months showed a waning posterior temporal response and by 10–13 months this response was negligible. Additional analysis showed that the age groups did not differ in their visual attention to the events and that changes in HbO were better explained by age in days than head circumference. In contrast to posterior temporal cortex, robust activation was obtained in occipital cortex across all ages tested. One interpretation of these results is that they reflect pruning of the visual object-processing network during the first year. The functional contribution of occipital and posterior temporal cortex, along with higher-level temporal areas, to infants' capacity to keep track of distinct entities in visual working memory is discussed.

## Introduction

Infants' capacity to track the identity of visual objects—to form coherent representations of objects that persist in the absence of direct visual input—changes substantially during the first year of life. Over the last 25 years developmental scientists have made significant progress toward understanding the nature and development of infants' capacity to represent objects in visual working memory. For example, investigations have revealed important changes in the type of information that infants include in their visual object representations, infants' capacity to integrate discordant sources of information, and the extent to which infants use this information to interpret physical events (Leslie et al., 1998; Tremoulet et al., 2000; Wilcox and Schweinle, 2002; Wang and Baillargeon, 2008; Baillargeon et al., 2012; Kaldy et al., 2015). There is also a growing body of research on the mechanisms that support and facilitate the changes that have been observed (Wang and Baillargeon, 2008; Wu et al., 2011; Baillargeon et al., 2012). One approach is to study the cognitive and cortical architecture on which the development of these capacities depends. With the introduction of more sophisticated neuroimaging and behavioral techniques that can be used with human infants in the experimental setting, the opportunities to apply a developmental cognitive neuroscience approach have expanded (Karmiloff-Smith, 2010; Wilcox and Biondi, 2015).

**Figure 1:**
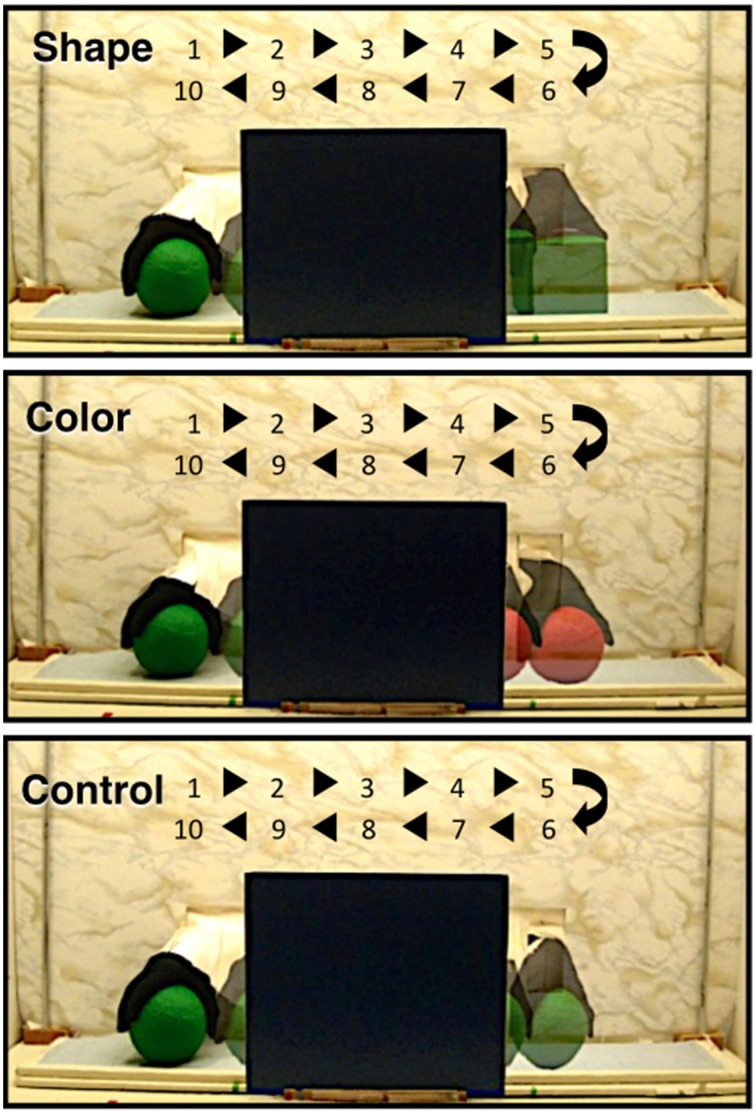
**The shape difference, color difference, and control test events used by Wilcox and her colleagues in previous studies (Wilcox et al., 2010, 2012, 2014a) and used in the present experiments**. The figure shows one event cycle. The numbers and arrows indicate the time (in s) and the space over which the objects moved during the event cycle. Infants saw 2 complete event cycles during each test trial.

**Figure 2:**
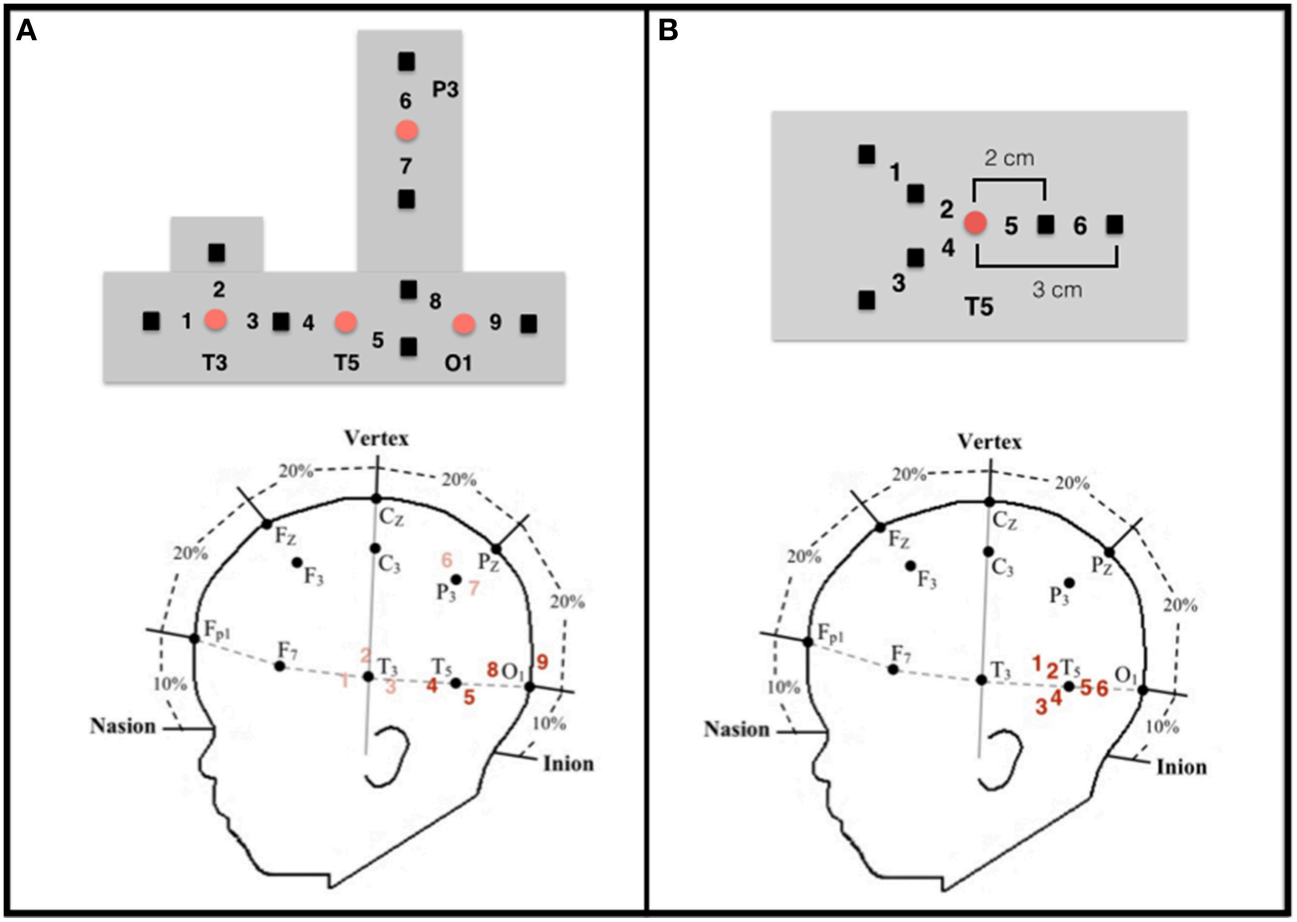
**Configuration and placement of optodes**. **(A)** Top: Configuration of the emitters (red circles) and detectors (black squares) and the nine corresponding channels in the headgear used by Wilcox et al. (2010, 2012, 2014a). Emitter-detector distances were all 2 cm. Bottom: Approximate location of the nine channels from which data were collected on a schematic of an infant's head in relation to the 10–20 International EEG system. Each detector read from a single emitter except for the detector between T3 and T5, which read from both emitters. The light was frequency modulated to prevent “cross-talk.” Experiment 1 focused on data collected at channels 4 and 5 (posterior temporal cortex) and channels 8 and 9 (occipital cortex), which are in bold. **(B)** Top: Configuration of the emitter (red circle) and detectors (black squares) and the six corresponding channels in the headgear used in Experiment 2. Emitter-detector distances were either 2 cm (channels 2, 4, and 5) or 3 cm (channels 1, 3, and 6). For statistical analyses (see text), the channels were grouped into three regions within the posterior temporal cortex: Region I (channels 1 and 2), Region II (channels 3 and 4), and Region III (channels 5 and 6).

**Figure 3:**
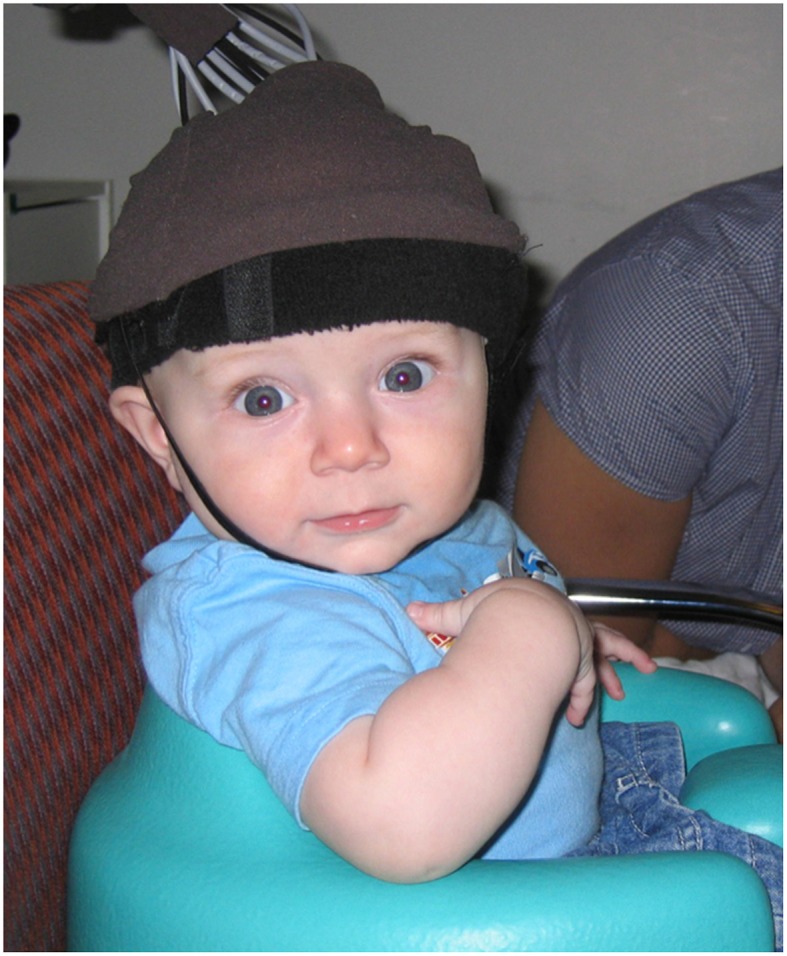
**An infant participant**. Infants sat in a supportive seat to restrain excess movement. An elasticized headband containing a rubberized piece in which the sources and detectors were embedded was slid onto the infant's head and secured by a chinstrap. Parental consent was obtained for use of the photograph for publication purposes.

In the adult, a number of cortical networks have been identified as important to visual object working memory. Of most interest to the current research are networks that support the processing of objects on the basis of their featural properties. Initial studies conducted with non-human primates, and subsequent studies conducted with adult humans, have revealed hierarchically organized networks in ventral areas of the cortex. For example, areas in the primary visual cortex respond to specific features, such as lines, orientation, or color (Bartels and Zeki, 2000; Tootell et al., 2003; Orban et al., 2004), whereas areas in the occipito-temporal cortex (e.g., lateral occipital complex) integrate these features and code objects as wholes, independent of visual perspective (Malach et al., 1995; Grill-Spector, 2003; Kanwisher, 2003; Kourtzi and Connor, 2011). Moving posterior to anterior in the temporal cortex, object representations become more abstract, with anterior temporal cortex being important to higher-level object processing, such as object identification and categorization (Humphreys et al., 1999; Devlin et al., 2002; Peelen and Caramazza, 2012). Recently, investigators have begun to explore the functional development of this network. In a series of studies, for example, infants aged 3–12 months were shown a shape-difference, color-difference, or control event like those depicted in Figure 1 (Wilcox et al., 2010, 2012, 2014a). Behavioral studies have revealed that early in the first year infants use the shape difference to individuate objects, but it is not until the end of the first year that infants use a color difference (Wilcox, 1999; Wilcox and Chapa, 2004). A similar developmental hierarchy has been observed in object segregation and identification tasks, which require related (but not identical) processes (Needham, 2000; Tremoulet et al., 2000). In the Wilcox et al. studies, functional near-infrared spectroscopy (fNIRS) was used to assess patterns of cortical activation during infants' processing of these events. Optodes were placed over three left ventral areas (Figures 2A, 3): occipital cortex (near O1 of the 10–20 International System for EEG recording), posterior temporal cortex (near T5 of the 10–20 System), and anterior temporal cortex (near T3 of the 10–20 System). Cortical activation was also measured from optodes placed over parietal cortex (near P3 of the 10–20 System) but this dorsal area is not of theoretical interest here. The main prediction was straightforward: a different pattern of activation would be obtained to events that engage, as compared to those that fail to engage, the individuation process. Whereas occipital cortex and posterior temporal cortex (low- and mid-level object processing areas) would be activated in response to all events, anterior temporal cortex (a higher level object processing area) would be activated only in response to events in which infants individuate by feature.

As predicted, these studies revealed consistent, robust activation in occipital cortex to all three events at all ages tested between 3 and 12 months (see also Wilcox et al., 2005, 2008, 2009). Also as predicted, anterior temporal activation was obtained only in response to events in which infants individuate-by-feature. For example, infants 3–9 months, who use shape but not color information to individuate objects (Wilcox, 1999; Wilcox and Chapa, 2004), showed activation in the anterior temporal cortex when viewing the shape difference but not the color difference event. In contrast, infants 11–12 months, who use shape and color information to individuate objects (Wilcox and Chapa, 2004; Wilcox et al., 2007) showed activation in the anterior temporal cortex when viewing the shape difference and the color difference event. The control event does not activate anterior temporal cortex in any age group. This pattern of results—activation only when infants interpret featural differences as signaling the presence of distinct objects—implicates the anterior temporal cortex as central to the individuation process. This conclusion is supported by evidence that when infants younger than 11 months (who do not spontaneously individuate-by-color) are shown events prior to test that prime them to individuate-by-color, activation in anterior temporal cortex is obtained (Wilcox et al., 2014b).

What was unexpected was the pattern of activation observed in posterior temporal cortex. Activation in this cortical area appears to be age-related and independent of test event. For example, infants about 3–6 months show activation in posterior temporal cortex in response to all three test events and the magnitude of the response does not vary by event (Wilcox et al., 2010, 2012). Other research has reported that activation in posterior temporal cortex in this age group is (a) specific to objects and not non-object visual stimuli such as reversing checkerboard patterns or faces (Watanabe et al., 2008, 2010; Lloyd-Fox et al., 2009; Honda et al., 2010), and (b) independent of the properties of the objects involved (Watanabe et al., 2008, 2010). Collectively, these data implicate the posterior temporal cortex as important to mid-level visual object processing (at least in younger infants); this area responds selectively to objects, although the information associated with those objects is limited. In contrast to the robust responses observed in younger infants, by about 7 months posterior temporal activation appears to wane and by about 12 months is typically not observed (Wilcox et al., 2012, 2014a), suggesting that the ventral object-processing network undergoes functional reorganization during the first year. Further exploration of this phenomenon is warranted, however, for a number of reasons. First, the studies from which these results are drawn used slightly different age groups, some of which overlap, hence firm conclusions about the age-related differences merits re-analysis of the data on the basis of age. Second, headgear configuration, including source-detector distances, remained constant across age while head circumference increased with age, raising questions as to the extent to which the change in HbO responses observed could be better explained by changes in head circumference (and hence the cortical areas being assessed) than age. Finally, only two measurement channels in posterior temporal cortex were used, leaving a portion of posterior temporal cortex un-assessed.

The goal of the current research was to assess the conclusion that activation in posterior temporal cortex during visual object processing wanes during the first year. The current research took two approaches. First, in Experiment 1 data from previously conducted studies in which infants saw shape-difference, color-difference, or control events were compiled into a single data set. Analyses of the responses at the two measurement channels in the posterior temporal cortex were conducted for each of three age groups: 3–6, 7–9, and 11–12 months. As a comparison, we analyzed hemodynamic responses at the two measurement channels in the occipital cortex, which have been reported to remain stable over the first year. In addition, we examined the correlation between hemodynamic responses, age, and head circumference, which has not been reported previously. Second, a new study was conducted in which optical imaging data were collected from six, rather than two, measurement channels in left posterior temporal cortex. Two age groups were tested in this experiment: 4- to 6-month-olds and 10- to 12-month-olds. This allowed us to assess the extent to which posterior temporal cortex might be involved in visual object processing in older infants, but had not been evident in previous studies because only two measurement channels were used.

## Experiment 1

The data for Experiment 1 were drawn from three previously published papers (Wilcox et al., 2010, 2012, 2014a) that had used a similar experimental protocol to assess activation in responses to the three events displayed in Figure 1. In these research reports, fNIRS data were collected while infants viewed a shape-difference, color-difference, or control event. We compiled the data from four measurement channels, two each in the occipital and posterior temporal cortex (Figure 2A), into a single database and hemodynamic responses along with age in days and head circumference. Although, all of these data were reported in previously published manuscripts, not all of the data were subjected to statistical analyses. For example, in Wilcox et al. (2014a) mean HbO responses in occipital and posterior temporal cortex were reported but not included in data analyses because they were not directly relevant to the research hypothesis. This approach allowed us to assess the extent to which responses in posterior temporal cortex, as compared to occipital cortex, differed by age when controlling for head circumference using a large sample.

### Materials and methods

#### Participants

The sample included 198 infants belonging to three age groups: 99–207 days (*n* = 93, *M* days = 170.5, 53 males, and 39 females); 213–280 days (*n* = 50, *M* days = 236.1, 32 males, and 18 females); and 339–391 days (*n* = 55, *M* days = 356.5, 34 males, and 21 females). These will be referred to as 3− to 6-month-olds (young age group), 7− to 9-month-olds (intermediate age group), and 11- to 12-month-olds (old age group), respectively. This sample included the infants tested in Wilcox et al. (2010) and Wilcox et al. (2012) and the 7- to 8-month-olds tested in Wilcox et al. (2014a)^1^. All data were collected using a between-subjects design. The number of infants who viewed the shape-difference, color difference, and control event in each age group was the following: young age group (shape *n* = 32, color *n* = 31, control *n* = 30), intermediate age group (shape *n* = 21, color *n* = 6, control *n* = 23), and old age group (shape *n* = 19, color *n* = 18, control *n* = 18). In each age group, an additional 22, 13, and 23 infants were tested, respectively, but excluded from analyses because of poor optical signal, failure to attend to the display, procedural problems, or crying. The percentage of infants who were tested but failed to contribute data did not differ significantly for the young (19.1%) and intermediate (20.6%) age groups, nor for the intermediate and old (29.5%) age groups, *p* > 0.05 (*Z*-test). The attrition rates reported in Experiment 1 and Experiment 2 are within the range of those typically reported in infant fNIRS studies (Lloyd-Fox et al., 2010). Experiment 1 and Experiment 2 were carried out in accordance with the recommendations and approval of the Institutional Review Board, Division of Research, Texas A&M University with written informed consent from the parents/guardians of all infant participants. All parents/guardians gave written informed consent in accordance with the Declaration of Helinski.

Infants were recruited from commercially produced lists, birth announcements in the local newspaper, and through social media. Parents were offered $5 or a lab T-shirt for participation. This study was carried out in accordance with the recommendations of the Institutional Review Board of Texas A&M University with written informed consent from parents of all participants. All parents gave written informed consent in accordance with the Declaration of Helsinki.

#### Task and procedure

Infants sat on their parent's lap or in a Bumbo® seat in a quiet and darkened room and watched the event to which they were assigned for four test trials, in a puppet-stage apparatus. Trained experimenters produced the test events live following a precise script. For the infants tested in Wilcox et al. (2010, 2012), test trials were 20 s in duration; for the infants tested in Wilcox et al. (2014a) test trials were 24 s in duration. Because analysis of the optical imaging data requires baseline recordings of the measured intensity of refracted light, each test trial was preceded by a 10 s baseline interval during which time a curtain covered the front opening and stage of the apparatus. The curtain was raised to begin each test trial.

Looking behavior was monitored by two independent observers who watched the infants through peepholes in cloth-covered frames attached to the side of the apparatus. Inter-observer agreement averaged 95% across all infants tested.

#### Instrumentation

The imaging equipment contained four fiber optic cables that delivered near-infrared light to the scalp of the participant (emitters), eight fiber optic cables that detected the diffusely reflected light at the scalp (detectors), and an electronic control box that served as the source of the near-infrared light and the receiver of the reflected light. The control box produced light at wavelengths of 690 nm, which is more sensitive to deoxygenated blood, and 830 nm, which is more sensitive to oxygenated blood, with two laser-emitting diodes (TechEn Inc). Laser power emitted from the end of the diode was 4 mW. Light was square wave modulated at audio frequencies of approximately 4–12 kHz. Each laser had a unique frequency so that synchronous detection could uniquely identify each laser source from the photodetector signal. Each emitter delivered both wavelengths of light and each detector responded to both wavelengths. The signals received by the control box were processed and relayed to a Dell desktop computer. A custom computer program recorded and analyzed the signal. Prior to test, infants were fitted with a custom-made headgear that secured the fiber optics to the scalp.

Configuration of the sources and detectors within the headgear, placement of the sources and detectors on the infant's head, and location of the measurement channels are displayed in Figure 2A. Source-detector separation was 2 cm. The headgear was not elastic so the distance between sources and detectors remained fixed. The headgear was placed on the infant's head using O1 as the anchor. For the purpose of this paper, we report only the data collected at O1 and T5. Head circumference of the infants tested ranged from 41 to 49 cm. Hence, the distance between O1 and T5 (1/5 of the head circumference) ranged from 8.2 to 9.8 cm. Although, head circumference did vary, the area of the skull (and underlying neural structures) affected was relatively small and, importantly, was smaller than the separation between each source and detector.

#### Processing of fNIRS data

The fNIRS data were processed, for each of the four detectors separately, using the same protocol (see Wilcox et al., 2010). Briefly, the raw signals were acquired at the rate of 200 samples per second, digitally low-pass-filtered at 10 Hz, a principal components analysis was used to design a filter for systemic physiology and motion artifacts, and the data were converted to relative concentrations of oxygenated (HbO) and deoxygenated (HbR) blood using the modified Beer-Lambert law. Changes in HbO and HbR were examined using the following time epochs: the 2 s prior to the onset of the test event, the 20 s (data from Wilcox et al., 2010, 2012) or 24 s (data from Wilcox et al., 2014a) test event, and the 10 s following the test event. The mean optical signal from −2 to 0 s (baseline) was subtracted from the signals and other segments of the time epoch were interpreted relative to this zeroed baseline.

Optical signals were averaged across trials and then infants for each event. Trials objectively categorized as containing motion artifacts (a change in the filtered intensity greater than 5% in 1.20 s during the 2 s baseline and test event) and in which infants failed to attend to the event were eliminated from the mean. These criteria eliminated 51 (of a possible 372), 41 (of a possible 200), and 56 (of a possible 220) trials in the young, intermediate, and old age groups, respectively. The percentage of missing trials was significantly greater for the intermediate than young age group, *z* = −2.108, *p* = 0.035 (two-tailed), but did not differ significantly for the intermediate and old age group, *z* = −1.203, *p* > 0.05. These data indicate that around 7–8 months it becomes more difficult for infants to successfully complete a full complement of test trials. It is interesting to note that whereas the age groups did not differ significantly in their attrition rates (reported in Section Participants), they did differ in the quantity of data that was collected within a test session. We suspect that once infants become independently mobile and can actively engage in reaching and object manipulation without trunk support, around 7 months of age, they become less cooperative in experiments that involve watching objects. This makes collection of fNIRS data, which is sensitive to motion artifacts, more challenging in older infants.

### Results and discussion

#### Looking time data

For each age group, duration of looking time data (in seconds) were averaged across trials and infants for each event and a One-way ANOVA was conducted with event as the between-subjects factor^2^. The main effect of event was not significant at any of the three age groups *(p* > *0.05)*. The mean (standard deviation) looking times of the young, intermediate, and old age groups were 16.52 s (2.90 s), 17.99 s (1.55 s), and 16.99 s (2.45 s).

#### Hemodynamic responses

For each age group, relative changes in HbO were averaged, for each event and channel, over 7–20 s (infants tested in Wilcox et al., 2010, 2012) or 7–24 s (infants tested in Wilcox et al., 2014a). This interval was chosen because the first emergence of the object to the right of the screen began at 5 s and, allowing 2 s for the hemodynamic response to become initiated, hemodynamic changes should be detectable by 7 s and persist until the end of the trial (see Wilcox et al., 2010 for supporting evidence). Statistical analyses are reported here for HbO responses only, which are more robust than HbR responses (Strangman et al., 2002). However, HbR data are reported in Supplementary Materials.

For each age group, preliminary analyses were conducted to assess the extent to which mean HbO responses could be explained by event or sex. In all analyses, no main effects or interactions involving these factors were obtained (*p* > 0.05). Hence, in subsequent analyses the data were collapsed across event and sex.

Two sets of analyses were performed on HbO responses. First, for each age group, mean responses obtained at channels 4 and 5 (posterior temporal cortex) and channels 8 and 9 (occipital cortex) were compared to 0. The outcome of these analyses, including Cohen's *d* effect sizes (Cohen, 1988), are reported in Table 1. For the young age group, a significant increase in HbO was obtained in both occipital channels (large effect sizes) and in both posterior temporal channels (large/medium effect sizes). For the intermediate age group, a significant increase in HbO was obtained in both occipital channels (large/medium effect sizes) and in both posterior temporal cortex channels (medium/small effect sizes). For the older infants, a significant increase in HbO was obtained in both occipital channels (large effect sizes) and in one posterior temporal channel (small effect size). In sum, significant activation, with medium to large effect sizes, was obtained in all occipital channels at all ages. In contrast, whereas strong activation was obtained in the posterior temporal cortex in the youngest age group it waned over time, and by 11–12 months only one channel showed activation and the magnitude of this response, as indicated by the effect size, was small and of little practical significance.

**Table 1:** **Mean (SD) HbO responses for the young, intermediate, and old age groups of Experiment 1**.

			**Young (3–6 Months)**	**Intermediate (7–9 Months)**	**Old (11–12 Months)**
Posterior Temporal (T5)	Channel 4	*M* (*SD*)	0.00497 (0.00644)	0.00374 (0.01046)	0.00198 (0.01089)
		*t* (*df*)	7.442 (92)	2.533 (49)	1.349 (54)
		*p*-value	< 0.001^***^	0.015^*^	0.183
		Cohen's *d*	1.104	0.523	0.257
	Channel 5	*M* (*SD*)	0.00329 (0.00650)	0.00246 (0.00818)	0.00194 (0.00622)
		*t* (*df*)	4.880 (92)	2.125 (49)	2.316 (54)
		*p*-value	< 0.001^***^	0.039^*^	0.024^*^
		Cohen's *d*	0.718	0.431	0.433
Occipital (O1)	Channel 8	*M* (*SD*)	0.00612 (0.00891)	0.00346 (0.00628)	0.00338 (0.00602)
		*t* (*df*)	6.625 (92)	3.899 (49)	4.171^***^ (54)
		*p*-value	< 0.001^***^	< 0.001^***^	< 0.001^***^
		Cohen's *d*	0.969	0.779	0.801
	Channel 9	*M* (*SD*)	0.00608 (0.00871)	0.00466 (0.00988)	0.00728 (0.01081)
		*t* (*df*)	6.734 (92)	3.355 (49)	4.994^***^ (54)
		*p*-value	< 0.001^***^	0.002^**^	< 0.001^***^
		Cohen's *d*	0.992	0.667	0.959

One sample t-tests were used to compare mean responses at each of the four channels, within the two cortical areas, to zero. Two-tailed p-values that passed the Benjamini and Hochberg (1995) test for multiple comparisons are indicated by asterisks:

* p < 0.05;

** p < 0.01;

*** p < 0.001.

A Cohen's d of 0.2, 0.5, and 0.8 are considered small, medium, and large effect sizes, respectively (Cohen, 1988).

**Figure 4:**
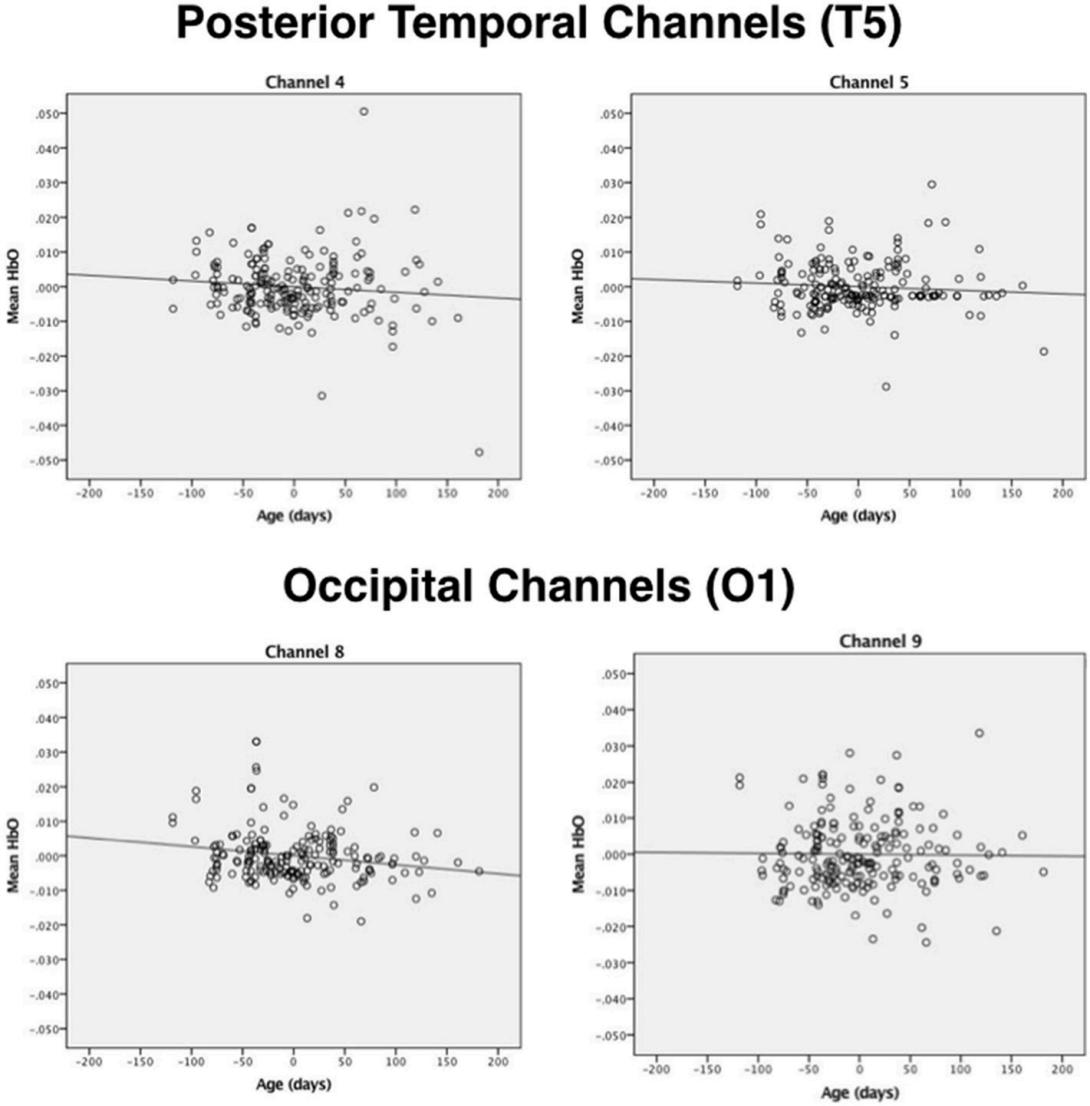
**Plots of the partial correlations for channels 4 and 5 (posterior temporal cortex) and channels 8 and 9 (occipital cortex) reported in Table 2**. The partial correlations obtained at channel 4 and channel 8 were significant after controlling for false discovery rates.

Next, correlational and partial correlational analyses were conducted to determine the relation between HbO responses at each of the four channels, age in days, and head circumference (HC). The correlational analyses (Table 2) revealed a significant negative correlation between age in days and HbO responses obtained in channels 4 and 8. Age in days and HC were positively correlated, as expected. Partial correlation analyses (Table 2) revealed that the negative correlation between age in days and HbO responses in channels 4 and 8 remained significant, even when controlling for HC. Plots of the partial correlations are displayed in Figure 4. These plots illustrate the fact that while the partial correlations were significant in channels 4 and 8, the effects sizes are relatively small. The negative correlation between age in days and HbO responses at channel 4 was predicted and is consistent with the group result reported above. The fact that age was not significantly, negatively correlated with HbO responses obtained in channel 5 was unexpected. This outcome suggests that HbO responses did not decrease linearly during the first year, but instead dropped exponentially at some point in time. The reported effect sizes (Table 1) suggest that the decline was greatest between the young and the intermediate age group, which is evident to some extent on the plots of the partial correlations. Finally, we were surprised by the negative correlation between age in days and HbO responses in channel 8. This outcome suggests that while occipital responses are robust at all ages, there may be subtle age-related changes that are not easily identifiable in smaller sample sizes. We will return to this is the General Discussion.

**Table 2:** **Correlation and partial correlation matrixes for Experiment 1**.

	**Age in days**	**Head circumference**	**Posterior temporal (T5)**		**Occipital (O1)**	
			**Channel 4**	**Channel 5**	**Channel 8**	**Channel 9**
**CORRELATIONS**						
Age in days	—	0.168	−0.128	−0.068	−0.145	0.076
*p*-value (one-tailed)	—	0.009^**^	0.036	0.17	0.021	0.144
Head Circumference	0.168	—	−0.056	−0.009	−0.054	−0.028
*p*-value (one-tailed)	0.009^**^	—	0.217	0.449	0.224	0.367
**PARTIAL CORRELATIONS**						
Age in days	—	—	−0.121	−0.068	−0.138	0.082
*p*-value (one-tailed)	—	—	0.046^*^	0.172	0.027^*^	0.127

Partial correlations controlled for head circumference. One-tailed p-values that passed the Benjamini and Hochberg (1995) test are indicated by asterisks:

* p < 0.05;

** p < 0.01;

^***^p < 0.001.

The results of Experiment 1 confirm that age-related changes in posterior temporal activation during visual object processing are marked and cannot be explained by changes in head circumference. As expected, age-related changes in occipital cortex were not evident in the group analyses and associated effect sizes; however, correlational analyses suggested that subtle age-related changes might exist. These findings will be discussed in more detail in the General Discussion. Experiment 2 was conducted to explore the extent to which age-related changes are observed in posterior temporal cortex when a larger array of channels is used.

## Experiment 2

Infants aged 4–6 and 10–12 months were presented with shape-difference, color-difference, and control events. These are all newly collected data. We focused on the younger and older infants because the change in activation in posterior temporal cortex is most pronounced between these two age groups. The headgear (Figure 2B) was designed to assess activation in posterior temporal cortex in areas nearby (but not identical to) the areas assessed in Experiment 1. Three of these channels had a source-detector distance of 2 cm and the other three channels had a source-detector distance of 3 cm. This allowed us to determine the extent to which activation was obtained at nearby areas, laterally and in depth, to those obtained in Experiment 1.

### Materials and methods

#### Participants

Infants aged 125–208 days (*n* = 18, *M* days = 168, 12 males, and 6 females) and aged 314–400 days (*n* = 16, *M* days = 352, 10 males, and 6 females) were tested. For ease in description these will be referred to as 4- to 6-month-olds (young age group) and 10- to 12-month-olds (old age group). All infants viewed shape-difference, color-difference, and control events. Given fewer measurement channels and improvements in headgear design, we were able to implement a within, rather than between, subject design. In the young and old age group an additional 8 and 13 infants were tested, respectively, but excluded from analyses because of poor optical signal, failure to attend to the display, procedural problems, or crying. The percentage of infants who were tested but failed to contribute data did not differ significantly for the young (30.8%) and old (38.5%) age groups, *p* > 0.05 (*Z*-test). The attrition rates observed in Experiment 2 are higher than those observed in Experiment 1, most likely due to the greater number of trials with which infants were presented (i.e., a lengthier experimental protocol). The race/ethnicity of the infants as reported by their parents was Caucasian (*n* = 29), Hispanic (*n* = 3), or mixed race/other (*n* = 2). Infants were recruited from commercially produced lists, birth announcements in the local newspaper, and social media websites. Parents were offered $5 or a lab T-shirt for participation. This study was carried out in accordance with the recommendations of the Institutional Review Board of Texas A&M University with written informed consent from parents of all participants. All parents gave written informed consent in accordance with the Declaration of Helsinki.

#### Task and procedure

The task and procedure were identical to that of Experiment 1 except that infants viewed all three events, for a total of 12 test trials (20 s each), rather than viewing one of the three events for 4 test trials. Infants saw the events in one of three randomly assigned orders (shape, control, color; control, shape, color; or color, shape, control). Inter-observer agreement averaged 91% across all infants tested.

#### Instrumentation

Instrumentation was similar to that of Experiment 1 with the exception of the headgear configuration (Figure 2B). One source, anchored at T5, and six detectors were used to create six measurement channels. Three of the detectors were placed 2 cm from the source and each of these had a corresponding detector placed 3 cm from the source, allowing for measurement at two cortical depths in three regions. The headgear was not elastic so the distance between the source and detectors remained fixed. The mean head circumference for the younger and older groups was 42.6 cm (*SD* = 1.31) and 46.6 cm (*SD* = 1.72), respectively. Hence, for the two age groups the mean difference in the distance between 01 and T5 (1/5 of the head circumference) was 0.8 cm.

#### Processing of fNIRS data

The fNIRS data were processed, for each detector separately, using a procedure similar to that of Experiment 1. Optical signals were averaged across trials and then infants for each event. Trials objectively categorized as containing motion artifacts and in which infants failed to attend to the event for at least 3 s were eliminated from the mean (We used a more liberal looking time criteria than in prior studies to increase data retention; this more liberal criteria did not alter the outcome of the HbO analyses). On the basis of these criteria, in the younger group 22 (of 208 possible) trials were eliminated from analysis and in the older group 54 (of 216 possible) trials were eliminated. The number of missing trials (in relation to total number of trials) differed significantly for the two age groups, *z* = −3.48 *p* < 0.0002 (two-tailed test). As in Experiment 1, the older and younger age groups did not differ significantly in attrition rates, but they did differ in the quantity of data collected within a test session.

### Results

#### Looking time data

For each age group, duration of looking time data (in seconds) were averaged across trials and infants for each event and a repeated measures One-way ANOVA was conducted with event as a within-subjects factor. The main effect of event was not significant for either age group (*p* > 0.05). The mean (standard deviation) looking times of the young and old age group were 15.77 s (2.13 s) and 16.14 s (2.82 s), respectively.

#### Hemodynamic responses

For each age group, relative changes in HbO were averaged, for each event and channel, over 7–20 s. Next, preliminary analyses were conducted to assess the extent to which mean HbO responses could be explained by event or sex. No main effects or interactions involving these factors were obtained (*p* > 0.05) in either age group. Hence, in subsequent analyses the data were collapsed across event and sex.

**Figure 5:**
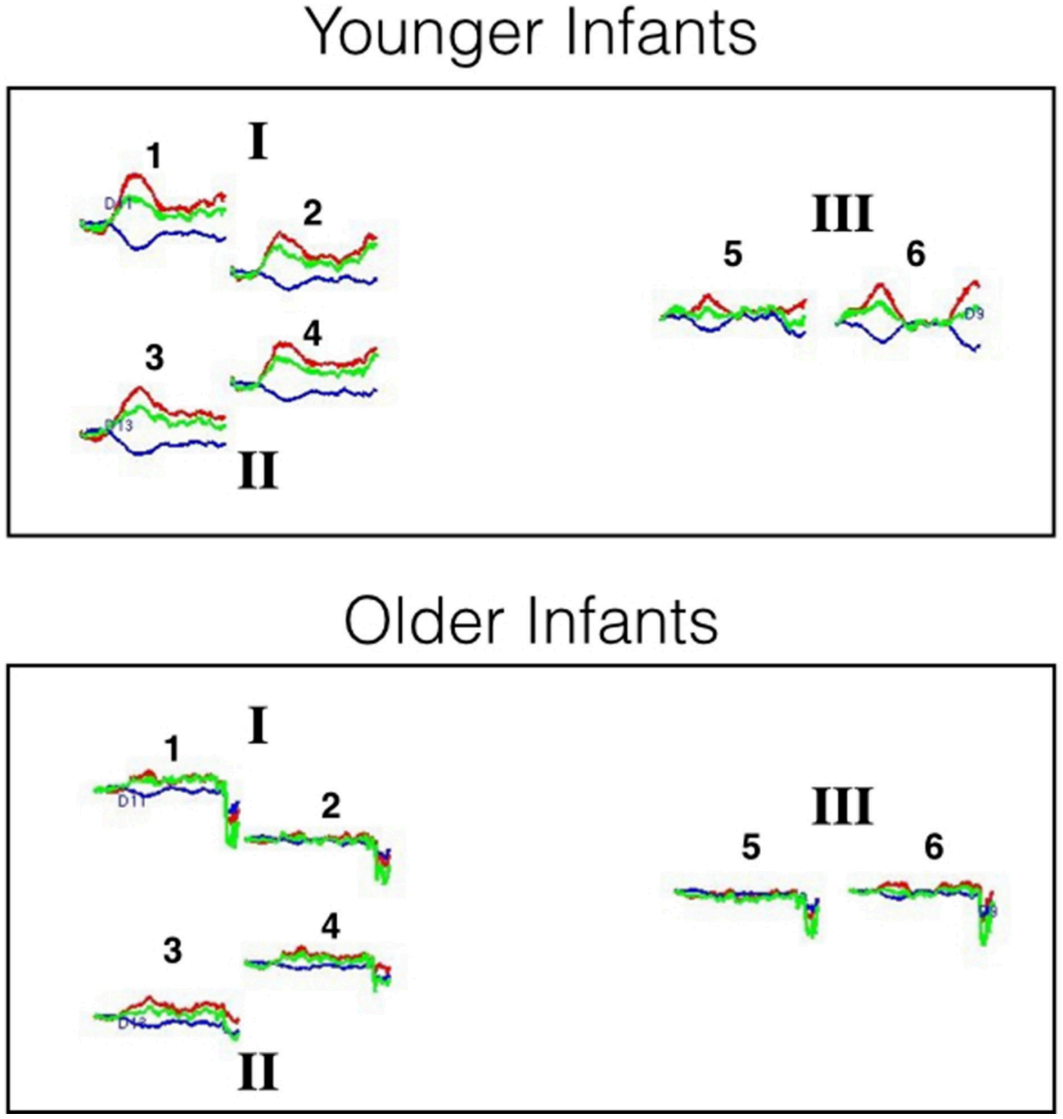
**Hemodynamic response curves for the younger and older infants of Experiment 2**. The number above each plot refers to the channel number and the Roman numerals refer to areas of interest. All regions are thought to lie within the posterior temporal cortex. The plot shows the mean HbO (red lines), HbR (blue lines), and HbT (green lines) curves in μM cm. Time is on the x-axis: the first black line on each plot denotes the onset of the 20 s test trial and the second black line denotes the onset of the 10 s baseline interval.

Preliminary analyses were also conducted to examine the extent to which HbO responses obtained at 2 and 3 cm source-detector distances differed. For each age group, mean responses obtained at each of the 6 channels (i.e., three pairs of channels, each pair including a 2 and 3 cm source-detector distance) in posterior temporal cortex were compared to 0 (see Supplementary Materials for HbO and HbR responses at each of the six channels). The outcome of these analyses indicate that very similar hemodynamic responses, with similar effect sizes, were obtained at channels 1 and 2, channels 3 and 4, and channels 5 and 6. This pattern held for both age groups. Hence, for the main analyses HbO data will be averaged across the two channels of each pair to create 3 regions of interest: I, II, and III, respectively. For illustrative purposes, the hemodynamic responses curves at each of the six channels, grouped into the three areas of interest, are displayed in Figure 5.

For each age group, mean responses obtained at each of the three ROIs were compared to 0. The outcome of these analyses (Table 3) revealed that for the young infants, a significant increase in HbO was obtained in all three ROIs, with medium to large effects sizes. For the older infants, no significant change in HbO was obtained in any of the ROIs. The effect sizes obtained with the older infants in Experiment 2 were equivalent or smaller than those obtained with the older infants in posterior temporal cortex in Experiment 1. These results confirm and extend those of Experiment 1 by revealing that across many different channels, young but not old infants show activation in posterior temporal cortex during visual object processing.

**Table 3:** **Mean (SD) HbO responses for the young and old age groups of Experiment 2**.

		**Young (4–6 months)**	**Old (10–12 months)**
Region I	*M* (*SD*)	0.0271 (0.0358)	0.0132 (0.0527)
	*t* (*df*)	3.211 (17)	1.002 (15)
	*p*-value	0.005^**^	0.332
	Cohen's *d*	1.065	0.354
Region II	*M* (*SD*)	0.0250 (0.0450)	0.0078 (0.0282)
	*t* (*df*)	2.135 (17)	1.111 (15)
	*p*-value	0.048	0.284
	Cohen's *d*	0.71	0.391
Region III	*M* (*SD*)	0.0150 (0.0312)	−0.0016 (0.0255)
	*t* (*df*)	2.038 (17)	−0.252 (15)
	*p*-value	0.057	0.804
	Cohen's *d*	0.68	0.089

One sample t-tests were used to compare mean responses at each of the three ROIs to zero. One-tailed p-values that passed the Benjamini and Hochberg (1995) test are indicated by asterisks: ^*^p < 0.05;

** p < 0.01;

^***^p < 0.001. Effect sizes as measured by Cohen's d are also reported Cohen (1988).

Correlation analyses for HC, age in days, and HbO responses were not conducted because HC and age in days (collapsed across the two age groups) were bi-modal in their distribution.

## General discussion

The research reported here clearly shows that younger and older infants demonstrate different patterns of activation in posterior temporal cortex during a visual object-processing task. Experiments 1 and 2, combined, revealed that when viewing moving, occluded objects infants aged 3–6 months show robust activation in posterior temporal cortex, measured at eight different channels surrounding T5, whereas infants aged 10–12 months showed little if any activation in any of these channels. Hemodynamic responses did not vary by event: regardless of the featural characteristics of the objects, and whether the same object or two different objects were seen to each side of the occluder, the same pattern of results was obtained. Additional data reported in Experiment 1 revealed that (a) an intermediate age group consisting of 7- to 9-month-olds showed HbO responses in posterior temporal cortex at a magnitude lesser than the young age group and greater than the old age group and (b) age-related changes in posterior temporal responses across the first year are better explained by age in days than head circumference.

The pattern of results obtained in the occipital cortex contrasted sharply with that obtained in posterior temporal cortex. Experiment 1 revealed strong hemodynamic responses in occipital cortex in all age groups (young, intermediate, old) to all test events. This outcome suggests that posterior temporal and occipital cortex play unique roles in visual object processing and, most relevant to the present discussion, that the contribution of posterior cortex to infants' processing of moving occluded objects changes considerably during the first year.

### Explaining age-related change in posterior temporal cortex

How do we interpret the age-related response obtained in posterior temporal cortex? One possibility is that these results reflect structural changes in the brain (e.g., increased density of neural tissue) or skull (e.g., increased skull thickness) that impede our ability to detect HbO responses. There are a number of reasons to question the viability of this explanation, the most notable being that activation has been obtained in posterior temporal cortex in infants older than 6 months (a) during these occlusion sequences but under different experimental conditions (Wilcox et al., 2014b) and (b) during other types of object processing tasks (Biondi and Wilcox, 2014, 2015). If structural changes interfere with our ability to measure activation in posterior temporal cortex, we would not expect to obtain responses in other experimental contexts. An alternative, and more likely, possibility is that these results reflect functional maturation of the ventral object-processing pathway. In the adult, ventral object processing networks are not only hierarchically organized, but also distributed in their organization. For example, processing of inanimate objects elicits activation in a distributed network of areas in the lateral occipital complex (LOC) and ventral temporal cortex (as well as intraparietal sulcus) and this pattern is distinct from that activated in response in animate objects (Haxby et al., 2001; Xu and Chun, 2006; Xu, 2009; Naughtin et al., 2014; Jacques et al., 2015). It is possible that object processing networks are not as discretely organized in the young infant, but become refined with time and experience. There are two lines of evidence that support the idea of functional pruning in ventral object processing areas. First, areas in the occipital cortex become more selective in their response to visual stimuli between 2 and 3 months of age; whereas some responses are widely distributed around 2 months they become localized to posterior areas of the occipital cortex by 3 months (Watanabe et al., 2008, 2010). Second, there is evidence from nonhuman primate studies that the neural pathway critical to visual object recognition memory, which projects from the inferior temporal cortex to medial temporal lobe structures, has an abundance of connections early in infancy. By adulthood, some connections are eliminated entirely or become more refined in their distribution (Webster et al., 1991; Bachevalier and Mishkin, 1994). These two examples, although drawn from cortical areas that mediate different object-processing functions in the ventral pathway, provide evidence for the importance of functional pruning during infancy. There are a number of mechanisms by which this pruning could occur, including intrinsic neurobiological factors, early experience with the external environment, and self-organizing principles that lead to select patterns of connectivity within and between cortical areas (Bachevalier and Hagger, 1991; Homae et al., 2010; Johnson, 2010; Kolb et al., 2014).

We are less sure of how to explain the negative correlation between age and the magnitude of the response obtained in occipital channel 8. We cannot rule out a “structural change” explanation as we did for the posterior temporal cortex. In our studies we typically obtain significant HbO responses in occipital cortex to all occlusion events at all ages tested; we have not observed age-related changes in response to these or related visual events. In addition, in Experiment 1 the negative relation between age in days and HbO revealed in the correlation and partial correlation analyses was not reflected in the group analyses: we obtained significant activation, with large effect sizes, in occipital channels at all age groups tested. These results suggest that the HbO responses observed in occipital cortex are so robust from an early age that a decline in the magnitude of the response over the first year does not lead to a qualitative change in the outcome of the statistical analyses. Since there is no evidence for functional pruning of occipital areas for the processing of these events, at least not at the ages tested, we favor a structural change explanation for the negative correlation we observed between age in days and HbO responses. In other words, we hypothesize that the negative correlation obtained in occipital cortex reflects a different process than that observed in posterior temporal cortex. Of course, further investigation is needed to test this hypothesis.

### Object processing and visual working memory

Arguably, infants' processing and interpretation of occlusion sequences like those used in the current experiments draws heavily on visual working memory. Infants must keep track of objects, and their unique numerical identities, as the objects move in and out of view behind the occluding screen. We know from previous work that anterior temporal cortex, in addition to occipital and posterior temporal cortex, plays a unique role in infants' processing of these events (Wilcox et al., 2010, 2012, 2014a). Activation is obtained in anterior temporal cortex in response to the occlusion sequences when the individuation process is engaged—when infants interpret the event as involving two numerically distinct objects. Activation is not obtained in anterior temporal cortex when the individuation process is not engaged. What is currently open to speculation is the specific processes mediated by these cortical areas: occipital cortex, posterior temporal cortex, and anterior temporal cortex. On the basis of what is currently known about adults' tracking of visual objects, we suspect that occipital cortex (and posterior temporal cortex in the younger infants) mediates short-term storage of occluded objects. For example, fMRI studies with adults have revealed that areas in LOC encode objects as whole entities rather than as parts (Malach et al., 1995; Grill-Spector, 2003; Kanwisher, 2003; Kourtzi and Connor, 2011), and are activated when feature sets change (Xu and Chun, 2006; Xu, 2009). However, it does not appear as though object features are bound to the objects as this stage in the processing (Xu, 2009; Naughtin et al., 2014). There is also evidence that LOC does not mediate the initiation and formation of distinct object representations, but is instead responsible for keeping track of already formed representations (Naughtin et al., 2014). Collectively, our data suggest that in the infant, anterior temporal cortex mediates the formation of distinct object representations, whereas the occipital cortex (and posterior temporal cortex in younger infants) is responsible for tracking those distinct entities through occlusion. The extent to which occipital areas are involved in infants' representation of feature sets, and the cortical basis of feature binding is open to debate. The charge of future research is to identify the ontogeny of cortical networks that support object representation, individuation, and identification. This endeavor will shed light on principles of brain development, such as the conditions under which networks are pruned, and can enhance our understanding of the cognitive architecture that supports acquisition of object knowledge during the first year.

## Author contributions

TW contributed to the conception and design of the work, data analysis and interpretation, and preparing the manuscript. MB contributed to data acquisition and interpretation, and aided significantly in preparation of the manuscript. TW and MB both agree to be accountable for the work and had final approval of the submitted version.

### Conflict of interest statement

The authors declare that the research was conducted in the absence of any commercial or financial relationships that could be construed as a potential conflict of interest.

## Footnotes

^1^Infants from Wilcox et al. (2010) and Wilcox et al. (2012) saw either a shape-difference, color-difference, or control event. However, only 17 infants from these two studies were aged 7–9 months. To increase the number of intermediate aged infants included in the sample, infants aged 7–9 months were also drawn from Wilcox et al. (2014a), for a final intermediate age samples size of n = 50. Infants in Wilcox et al. (2014a) were not presented with a color-difference event, hence, most infants in the intermediate group saw either a shape-difference or control event.

^2^Recall that the infants in the intermediate age group who were drawn from Wilcox et al. (2014a) had a test trial length of 24 s rather than 20 s. The looking time data of these infants was adjusted for trial length (duration of looking × 0.833).
