# Corrigendum: Functional Activation in the Ventral Object Processing Pathway during the First Year

**DOI:** 10.3389/fnsys.2016.00038

**Published:** 2016-05-03

**Authors:** Teresa Wilcox, Marisa Biondi

**Affiliations:** Infant Cognition Lab, Department of Psychology, Texas A&M UniversityCollege Station, TX, USA

**Keywords:** infants, object processing, object processing pathway, ventral temporal cortex, cortical development

Table 3 of the Wilcox and Biondi (2016) article contained the incorrect *p*-values, which we hereby rectify. The original table contained two-tailed *p*-values rather than one-tailed *p*-values. The legend of Table 3 indicated that one-tailed values were reported. We therefore re-submit Table 3 with the correct one-tailed *p*-values.

**Table 3:** **Mean (SD) HbO responses for the young and old age groups of Experiment 2**.

**Areas of Interest**		**Young (4–6 months)**	**Old (10–13 months)**
Region I	*M* (*SD*)	0.0271(0.0358)	0.0132(0.0527)
	*t* (*df*)	3.211 (17)	1.002 (15)
	*p*-value	0.0025^**^	0.116
	Cohen's *d*	1.065	0.354
Region II	*M* (*SD*)	0.0250(0.0450)	0.0078(0.0282)
	*t* (*df*)	2.135 (17)	1.111 (15)
	*p*-value	0.024^**^	0.142
	Cohen's *d*	0.71	0.391
Region III	*M* (*SD*)3	0.0150(0.0312)	–0.0016 (0.0255)
	*t* (*df*)	2.038 (17)	–0.252 (15)
	*p*-value	0.0285^**^	0.402
	Cohen's *d*	0.68	0.089

One sample t-tests were used to compare mean responses at each of the three ROIs to zero. One-tailed p-values that passed the Benjamini-Hochberg (Benjamini and Hochberg, 1995) test are indicated by asterisks: ^*^ p < 0.05;

** p < 0.01;

^***^ p < 0.001. Effect sizes as measured by Cohen's d are also reported (Cohen, 1988).

## Author contributions

TW contributed to the conception and design of the work, data analysis and interpretation, and manuscript preparation. MB contributed to data acquisition and interpretation, and manuscript preparation.

### Conflict of interest statement

The authors declare that the research was conducted in the absence of any commercial or financial relationships that could be construed as a potential conflict of interest.
