# Supplementary material for: Functional Activation in the Ventral Object Processing Pathway during the First Year
Source: Front Syst Neurosci. 2016 Jan 5;9:180. doi: 10.3389/fnsys.2015.00180 (PMC4700261; doi:10.3389/fnsys.2015.00180)
Supplement: Supplementary file 1 [file DataSheet1.docx]

**Appendix A**

|  |  |  | **Young**  **(3-6 Months)** | **Intermediate**  **(7-9 Months)** | **Old**  **(11-12 Months)** |
| --- | --- | --- | --- | --- | --- |
| **Posterior Temporal (T5)** | **Channel 4** | ***M* (*SD*)** | -.00083 (.00287) | -.00045 (.00486) | .00004 (.00395) |
|  |  | ***t* (*df*)** | -2.966 (92) | -.651 (49) | .067 (54) |
|  |  | ***p*-value** | **.004**** | 0.518 | 0.947 |
|  |  | **Cohen’s *d*** | -0.409 | -0.1309 | -0.0143 |
|  | **Channel 5** | ***M* (*SD*)** | -.00124 (.00295) | -.00058 (.00362) | .00002 (.00196) |
|  |  | ***t* (*df*)** | -4.038 (92) | -1.123 (49) | .075 (54) |
|  |  | ***p*-value** | **<.001***** | 0.267 | 0.94 |
|  |  | **Cohen’s *d*** | -0.5944 | -0.2265 | 0.0144 |
| **Occipital (O1)** | **Channel 8** | ***M* (*SD*)** | -.00098 (.00215) | -.00108 (.00181) | -.00083 (.00188) |
|  |  | ***t* (*df*)** | -4.376 (92) | -4.193 (49) | -3.288 (54) |
|  |  | ***p*-value** | **<.001***** | **<.001***** | **.002**** |
|  |  | **Cohen’s *d*** | -0.6446 | -0.8438 | -0.6244 |
|  | **Channel 9** | ***M* (*SD*)** | -.00108 (.00289) | -.00188 (.00349) | -.00135 (.00344) |
|  |  | ***t* (*df*)** | -3.607 (92) | -3.807 (49) | -2.906 (54) |
|  |  | ***p*-value** | **.001***** | **<.001***** | **.005**** |
|  |  | **Cohen’s *d*** | -0.5285 | -0.7618 | -0.555 |

**Appendix B**

|  |  |  | **Mean HbO (SD)** | | **Mean HbR (SD)** | |
| --- | --- | --- | --- | --- | --- | --- |
|  |  |  | **Young**  **(4-6 Months)** | **Old**  **(10-12 Months)** | **Young**  **(4-6 Months)** | **Old**  **(10-12 Months)** |
| **Region I** | **Channel 1** | ***M* (*SD*)** | .01172 (.02788) | .00521 (.02552) | -.00708 (.01906) | -.00429 (.00934) |
|  |  | ***t* (*df*)** | 1.784 (17) | -.817 (15) | -1.577 (17) | -1.838 (15) |
|  |  | ***p*-value** | 0.092 | 0.427 | 0.133 | 0.086 |
|  |  | **Cohen’s *d*** | 0.5931 | 0.2884 | -0.5261 | -0.5253 |
|  | **Channel 2** | ***M* (*SD*)** | .01821 (.03962) | .00200 (.02718) | -.00384 (.01744) | -.00472 (.01359) |
|  |  | ***t* (*df*)** | 1.950 (17) | .295 (15) | -.934 (17) | -1.389 (15) |
|  |  | ***p*-value** | 0.068 | 0.772 | 0.364 | 0.185 |
|  |  | **Cohen’s *d*** | 0.655 | 0.104 | -0.3114 | -0.4912 |
| **Region II** | **Channel 3** | ***M* (*SD*)** | .02184 (.04481) | .00282 (.02766) | -.00872 (.01518) | -.00243 (.01049) |
|  |  | ***t* (*df*)** | 2.068 (17) | .408 (15) | -2.436 (17) | -.926 (15) |
|  |  | ***p*-value** | 0.054 | 0.689 | 0.026 | 0.369 |
|  |  | **Cohen’s *d*** | 0.6892 | 0.1435 | -0.8124 | -0.3276 |
|  | **Channel 4** | ***M* (*SD*)** | .02825 (.05807) | .01286 (.03035) | -.01246 (.02441) | -.00448 (.01417) |
|  |  | ***t* (*df*)** | 2.064 (17) | 1.695 (16) | -2.165 (17) | -1.266 (15) |
|  |  | ***p*-value** | 0.055 | 0.111 | 0.045 | 0.225 |
|  |  | **Cohen’s *d*** | 0.6734 | 0.6002 | -0.7219 | -0.4472 |
| **Region III** | **Channel 5** | ***M* (*SD*)** | .02733 (.03390) | .01101 (.05413) | -.00797 (.01893) | -.00489 (.02559) |
|  |  | ***t* (*df*)** | 3.420 (17) | .817 (15) | -1.787 (17) | -.764 (15) |
|  |  | ***p*-value** | **.003**** | 0.427 | 0.092 | 0.456 |
|  |  | **Cohen’s *d*** | 1.1401 | 0.2889 | -0.5954 | -0.2702 |
|  | **Channel 6** | ***M* (*SD*)** | .02683 (.04099) | .01534 (.05235) | -.01354 (.02213) | -.00955 (.02347) |
|  |  | ***t* (*df*)** | 2.777 (17) | 1.172 (15) | -2.595 (17) | -1.627 (15) |
|  |  | ***p*-value** | **.013*** | 0.26 | 0.019 | 0.125 |
|  |  | **Cohen’s *d*** | 0.9257 | 0.4144 | -0.8653 | -0.5754 |

**Appendix Legends**

**Appendix A.** Mean (SD) HbR responses for the young, intermediate, and old age groups of Experiment 1. One sample t-tests were used to compare mean responses at each of the four channels, within two cortical areas, to zero. Two-tailed *p*-values that passed the Benjamini-Hochberg (Benjamini & Hochberg, 1995) test for multiple comparisons are indicated by asterisks: * *p* < .05; ** *p* < .01; *** *p* < .001. Effect sizes as measured by Cohen's *d* are also reported (Cohen, 1988).

**Appendix B*.*** Mean (SD) HbO and HbR responses for each of the six channels, thought to lie within the posterior temporal cortex, by age group, of Experiment 2. One sample t-tests were used to compare mean responses at each channel to zero. One-tailed *p*-values that passed the Benjamini-Hochberg (Benjamini & Hochberg, 1995) test are indicated by asterisks: * *p* < .05; ** *p* < .01; *** *p* < .001. Effect sizes as measured by Cohen's *d* are also reported (Cohen, 1988).
